# Inhibition of HCN1 currents by norquetiapine, an active metabolite of the atypical anti-psychotic drug quetiapine

**DOI:** 10.3389/fphar.2024.1445509

**Published:** 2024-10-07

**Authors:** Amélie Jean Jacques, Nazzareno D’Avanzo

**Affiliations:** Département de Pharmacologie et Physiologie, Université de Montréal, Montréal, QC, Canada

**Keywords:** HCN channel, norquetiapine, quetiapine, depression, major mood disorder

## Abstract

Quetiapine is a second-generation atypical antipsychotic drug that has been commonly prescribed for the treatment of schizophrenia, major depressive disorder (depression), and other psychological disorders. Targeted inhibition of hyperpolarization-activated cyclic-nucleotide gated (HCN) channels, which generate I_h_, may provide effective resistance against schizophrenia and depression. We investigated if HCN channels could contribute to the therapeutic effect of quetiapine, and its major active metabolite norquetiapine. Two-electrode voltage clamp recordings were used to assess the effects of quetiapine and its active metabolites 7-hydroxyquetiapine and norquetiapine on currents from HCN1 channels expressed in *Xenopus laevis* oocytes. Norquetiapine, but not quetiapine nor 7-hydroxyquetiapine, has an inhibitory effect on HCN1 channels. Norquetiapine selectively inhibited HCN1 currents by shifting the voltage-dependence of activation to more hyperpolarized potentials in a concentration-dependent manner with an IC_50_ of 13.9 ± 0.8 μM for HCN1 and slowing channel opening, without changing the kinetics of closing. Inhibition by norquetiapine primarily occurs from in the closed state. Norquetiapine inhibition is not sensitive to the external potassium concentration, and therefore, likely does not block the pore. Norquetiapine inhibition also does not dependent on the cyclic-nucleotide binding domain. Norquetiapine also inhibited HCN4 channels with reduced efficacy than HCN1 and had no effect on HCN2 channels. Therefore, HCN channels are key targets of norquetiapine, the primary active metabolite of quetiapine. These data help to explain the therapeutic mechanisms by which quetiapine aids in the treatment of anxiety, major depressive disorder, bipolar disorder, and schizophrenia, and may represent a novel structure for future drug design of HCN inhibitors.

## Introduction

Hyperpolarization-activated cyclic-nucleotide gated (HCN) channels are the molecular correlate of I_h_, and are widely expressed in the central and peripheral nervous systems. All four isoforms (HCN1-4) are expressed in the brain (Ludwig et al., 1998; Moosmang et al., 2001; Pape, 1996; Santoro et al., 1997; Santoro et al., 1998) where they play a role in setting the resting membrane potential, modulating dendritic integration of synaptic inputs, reducing neuronal input resistance, neuronal pacemaking, and establishing action potential threshold (Pape, 1996). HCN channels are important for learning and memory, pain sensation, sour taste sensation, and vision (Chaplan et al., 2003; Knop et al., 2008; Nolan et al., 2004; Nolan et al., 2003; Paspalas et al., 2013; Stevens et al., 2001). HCN1^−/−^ mice show impaired motor learning but enhanced spatial learning and memory (Nolan et al., 2004; Nolan et al., 2003) and enhanced resistance to depression (Lewis et al., 2011; Huang et al., 2009). HCN1 expression increases in the CA1 region of the dorsal hippocampus in a chronic unpredictable stress rat model. Notably, shRNA knockdown of HCN1 reduces the stress response in this model (Kim et al., 2018). Targeted viral knockdown HCN1 in the CA1 hippocampal region also enhanced mobility in the Porsolt swim test (Kim et al., 2012). Similarly, genetic ablation of *Trip8b*, an auxiliary protein that regulates HCN1 and HCN2 expression, also increases resistance to depression (Lewis et al., 2011). Furthermore, altered HCN-cAMP signaling in prefrontal cortex networks also appears to contribute to the working memory deficits in schizophrenia and stress (Paspalas et al., 2013; Arnsten, 2011; Gamo et al., 2015), while mutations in SHANK3 linked to schizophrenia (Gauthier et al., 2010; Guilmatre et al., 2014) may induce an HCN channelopathy (Yi et al., 2016). Furthermore, polymorphisms in HCN4 channels were associated with mood disorders and/or obsessive compulsive disorder (Szabo et al., 2011).

**FIGURE 1:**
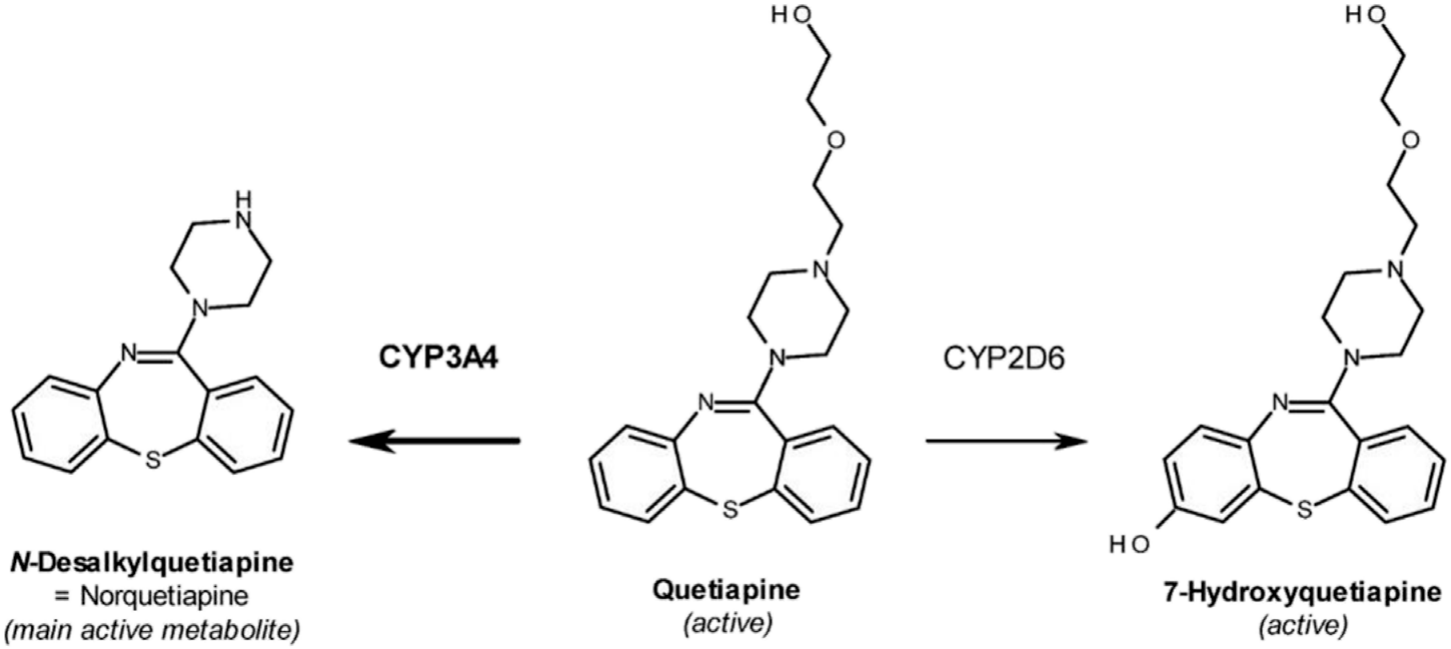
Chemical structures of Quetiapine (QTP) and its active metabolites Norquetiapine (NQTP) and 7-hydroxyquetiapine (7-OH QTP).

Quetiapine fumarate (Seroquel^®^) (QTP) (Figure 1) is a second-generation atypical antipsychotic drug that has been commonly prescribed for the treatment of schizophrenia (Small et al., 1997; Dev and Raniwalla, 2000), acute bipolar mania (Janicak and Rado, 2012), insomnia (Lin et al., 2023), major depressive disorder (depression) (Ravindran et al., 2022), anxiety (Ravindran et al., 2022; Crapanzano et al., 2021; Bandelow et al., 2010), Post-traumatic stress disorder (Crapanzano et al., 2023) and other psychological disorders (Saller and Salama, 1993). Like other atypical antipsychotics, QTP is structurally similar to clozapine and acts as an antagonist to serotonin, dopamine, histamine, and adrenergic receptors (Saller and Salama, 1993; Burns, 2001). QTP is primarily metabolized by hepatic cytochrome P450 3A4 (Dev and Raniwalla, 2000), with norquetiapine (NQTP) and 7-hydroxyquetiapine (7-OH QTP) as its major active metabolites (Figure 1). NQTP exhibits pharmacological activity that differs from QTP (Bakken et al., 2012; DeVane and Nemeroff, 2001) and also exhibits antidepressant activity (Jensen et al., 2008; Lopez-Munoz and Alamo, 2013). In fact, NQTP shares structural similarities with several antidepressants including amoxapine and desipramine, and its physicochemical properties confer greater potential for its use as an antidepressant agent (Lopez-Munoz and Alamo, 2013; Kim et al., 2016). Indeed, the effect of QTP in major depressive disorder is probably mediated, at least in part, by NQTP, which selectively inhibits norepinephrine transporter reuptake (Bandelow et al., 2010; Lopez-Munoz and Alamo, 2013).

In addition to antagonizing serotonin, dopamine, histamine, and adrenergic receptors, and inhibiting norepinephrine transporter reuptake, QTP and NQTP block the hERG (human-Ether-a-go-go-Related Gene) potassium channel (Kongsamut et al., 2002; Lee et al., 2018), and the sodium channel, Nav1.5 (Kim et al., 2020). Given the role of HCN channels in major depressive disorders, anxiety, and schizophrenia, examining the effects of antipsychotic drugs on this current is worthwhile. In the present study, we investigated the inhibitory mechanisms of norquetiapine on the HCN channels expressed in *Xenopus laevis* oocytes.

## Methods

### Cloning, oocyte isolation and channel expression

cDNA coding for the mouse HCN1 gene and mHCN1-CX5 containing a stop codon at residue F472 (denoted as HCN1-ΔCNBD in this article) was sub-cloned into the expression vector pGH19, while mouse HCN2 was sub-cloned into the pGEM vector. hHCN4 was expressed in pcDNA3.1. All clones were verified by PCR sequencing of the complete ORF. cDNA was linearized using NheI (New England Biolabs) for mHCN1, SphI for mHCN2, or XbaI for hHCN4. To obtain RNA, ∼1.0 μg of linearized cDNA was used for *in vitro* transcription synthesis using the mMESSAGE mMACHINE™ T7 Transcription kit (Thermo Fisher Scientific, Life Technologies, United States).

All experiments were preformed using unfertilized *Xenopus* oocytes, extracted from anaesthetized female *X. laevis*. Once extracted, oocytes were injected with 4.6 ng of HCN RNA using a Drummond Nanoject II injector (Drummond Scientific Company). Prior to injection oocytes were subject to a controlled temperature of 17°C–19°C and placed in vials containing Barth antibiotic solution (mM): 90 NaCl, 3 KCl, 0.82 MgSO_4_.7H_2_O, 0.41 CaCl_2_.2H_2_O, 0.33 Ca(NO_3_)_2_.4H_2_O and 5 HEPES supplemented with 100 U/mL of penicillin-streptomycin and 10 mg/mL of kanamycin stock (10 mg/mL). Post injection cells were incubated in Barth antibiotic serum solution supplemented with ∼5% horse serum. Cells were expressed and ready to be used in electrophysiological recordings 1–3 days post injection. The number of cells recorded for each experimental group (n) are presented. Data for each group were collected from oocytes harvested on at least 3 separate occasions. Data subjected to statistical analysis had an n of at least five for each group.

### Electrophysiological recordings

Electrophysiological studies were conducted using the two-electrode voltage clamp (TEVC) technique. Borosilicate rapid fill microelectrode pipettes (FHC Inc., United States) were filled with filtered 1 M KCL solution. Macroscopic currents were recorded from oocytes expressing full-length HCN1, HCN2, HCN4, or HCN1-ΔCNBD in a bath solution containing (in mM): 5 KCl, 84 NaCl 15 HEPES, 0.4 CaCl2, and 0.8 MgCl2, pH = 7.4 using OC-725C amplifier (Warner Instruments, United States) and digitized using a Digidata 1322 A (Molecular Devices, Sunnyvale, CA, United States). High [K^+^] recordings contained 30 mM KCl, and 59 mM NaCl instead. Quetiapine (QTP), 7-hydroxyquetiapine (7-OH QTP) or norquetiapine (NQTP) (Toronto Research Chemicals, Toronto, ON, Canada) were dissolved in DMSO to make 100 mM stock solutions that were stored at −20°C. On the day of the experiments, the stock solutions were diluted in extracellular solution to the final desired concentrations. All data were acquired using the software Clampex 10.5 at a sampling rate of 5 KHz with a filter of 1 kHz. HCN1 activation was assessed by 1.7 s test-steps between −130 and −30 mV (ΔV = +10 mV) from a V_H_ = −30 mV, followed by a 1.5 s step to −130 mV. Deactivation was assessed by applying a 1.75 sec pre-pulse to −130 mV, followed by test pulses from +50 to −70 mV (ΔV = −10 mV). Since HCN2 channels activate slower and at more negative potentials, the protocol was altered to 2 s test-steps between −160 and −20 mV (ΔV = +10 mV) from a V_H_ = −30 mV, followed by a 1.5 s step to −160 mV. Similarly, HCN4 were activate by 3 s test-steps between −160 to −20 mV (ΔV = +10 mV) from a V_H_ = −30 mV, followed by a 1 s step to −160 mV. In all recordings, cells were held at the holding potential for an inter-pulse time of 27 s to allow the channels to fully close between sweeps. Control recordings (0 µM) were performed 2 min after impaling the cells, to allow stabilization of currents, and then QTP, 7-OH QTP or NQTP were added to the bath solution for at the defined concentration for 7.5 min (15 or 30 min in some cases as indicated) to enable pair-wise experiments. Experiments were also performed with equimolar quantities of DMSO used to solvate the drugs to their listed concentrations were used as additional controls. All compounds were tested for up to 30 min to ensure any effects or lack thereof were not time dependent. All recordings were conducted at room temperature (20–23°C).

Open-state inhibition was assessed using a constant pulse to −130 mV and adding 30 µM NQTP once HCN1 currents reached steady-state. Closed state inhibition was assessed using a repetitive pulse protocol with 2s pulses to −130 mV every 30 s from a holding potential (V_H_) of −10 mV. Inhibition by 30 µM NQTP was compared when repetitive pulses were continuously applied during NQTP addition, or cells were held at V_H_ for 7.5 min.

### Data analysis and statistics

All recordings were analyzed offline using the Clampfit (Molecular Devices) software. Data was analyzed and plotted using Origin 8.0 software (Northampton, MA, United States). Current-voltage relationships were analyzed using built in software in pClamp, taking each respective voltage to an inquired current. The I-V relationship was fit with the Boltzman I-V equation:

$$I=\frac{\left(V_{m}-V_{rev}\right)g_{\max}}{1+e^{\frac{V_{m}-V_{\frac{1}{2}}}{k}}}$$ (1)

Activation and deactivation kinetics were determined with mono- or bi-exponential fits of test pulses after the initial lag period, as shown. Steady-state activation curves were fit with the Boltzmann equation:

$$G/G_{\mathrm{Max}}=\frac{1}{1+e^{\frac{V_{m}-V_{\frac{1}{2}}}{k}}}$$ (2)

where V_m_ corresponds to the test pulse, V_1/2_ is the midpoint of activation and k is the inverse slope factor. Concentration dependences of the drug-induced shift in V_1/2_ (ΔV_1/2_) were fit with the Hill equation:

ΔV12ΔV12max=11+ IC50drugnH (3)

where nH is the Hill co-efficient. Data are presented as means (±) standard error of the total number of cells (*N*). Statistical significance for I-V curves were determined measured using two-way ANOVA with Tukey HSD *post hoc* analysis. V_1/2_’s of steady-state dependencies were determined for each recording and pooled for a given treatment then analyzed by pairwise student t-test. A P value <0.05 was considered as statistically significant.

## Results

### Inhibition of HCN1 channel by norquetiapine but not quetiapine

**FIGURE 2:**
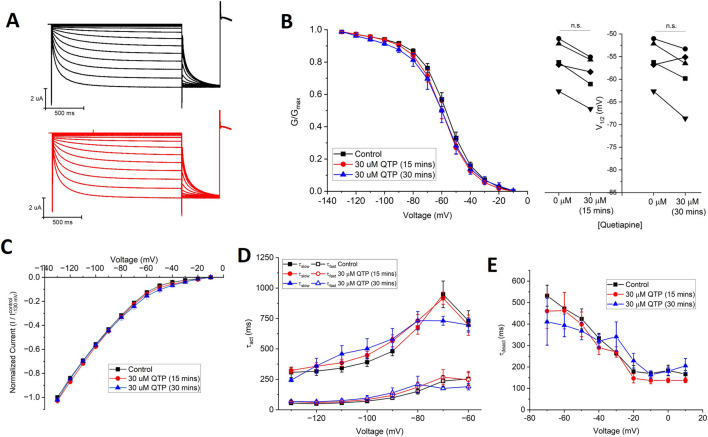
Quetiapine (QTP) does not regulate HCN1 channels. **(A)** Representative traces from a paired activation experiment following the addition of 30 µM QTP to oocytes expressing full-length HCN1. 30 μM QTP does not affect **(B)** the voltage-dependence of activation (V_1/2_ of each cell is shown on the right) (n = 5, P = 0.11) **(C)** the current-voltage (I-V) relationship (n = 5, P = 0.35) **(D)** activation kinetics (n = 5, P = 0.56) or **(E)** deactivation kinetics (n = 5, P = 0.72) of HCN1 channels.

Since 30 µM quetiapine (QTP) induces ∼80% inhibition of hERG channels (Lee et al., 2018) and 50% inhibition of Nav1.5 channels (Kim et al., 2020), we examined the effects of 30 µM QTP on HCN1 channels expressed in *X. laevis* oocytes using TEVC. At this concentration, in paired experiments, we observed no significant changes in the current-voltage relationship, steady-state voltage-dependence, nor gating kinetics of HCN1 (Figure 2, Tables 1, 2) using Equations 1, 2. No effect of QTP on HCN1 is observable, even if the incubation period is extended from 15 min to 30 min (Figure 2).

**TABLE 1:** Voltage-dependence of activation in the presence and absence of QTP or NQTP.

Pair-wise conditions	V ½ (mV)	k	n	P-value for V_1/2_
HCN1 Control	−56.4 ± 4.4	10.3 ± 0.8	5	
HCN1 30 µM QTP	−59.3 ± 2.1	10.3 ± 0.3	5	0.11
HCN1 Control	−60.2 ± 1.4	10.6 ± 1.2	10	
HCN1 30 µM NQTP	−72.0 ± 2.1	10.9 ± 1.8	10	<0.001
HCN1 Control	−59.0 ± 1.6	10.4 ± 0.5	5	
HCN1 7-OH QTP	−59.2 ± 0.9	11.1 ± 0.7	5	0.86
HCN2 Control	−93.0 ± 1.1	11.1 ± 0.5	6	
HCN2 30 µM NQTP	−96.3 ± 1.2	10.6 ± 0.7	6	0.09
HCN4 Control	−104.6 ± 1.6	13.8 ± 0.9	9	
HCN4 30 µM NQTP	−111.1 ± 1.4	15.8 ± 1.1	9	0.001
HCN1 30 mM [K^+^]_o_ Control	−60.9 ± 3.4	13.3 ± 2.3	6	
HCN1 30 mM [K^+^]_o_ 30 µM NQTP	−72.7 ± 6.2	11.7 ± 2.4	6	<0.01
HCN1ΔCNBD Control	−74.8 ± 2.0	13.7 ± 0.5	6	
HCN1ΔCNBD 30 µM NQTP	−84.3 ± 3.0	12.2 ± 0.9	6	<0.01

**TABLE 2:** Kinetics of activation in the presence and absence of QTP or NQTP.

Pair-wise conditions	τ_act_ fast (ms)	τ_act_ slow (ms)	P-value
HCN1 Control (−100 mV)	412 ± 24	75 ± 7	
30 µM QTP 15 min (−100 mV)	405 ± 59	78 ± 12	0.22
30 µM QTP 30 min (−100 mV)	469 ± 51	94 ± 14	0.19
HCN1 Control (−100 mV)	482 ± 38	102 ± 8	
30 µM NQTP 7.5 min (−100 mV)	784 ± 75	179 ± 16	<0.05
30 µM NQTP 15 min (−100 mV)	728 ± 104	169 ± 23	<0.05
HCN1 Control	417 ± 25	81 ± 4	
7-OH QTP (−100 mV)	453 ± 46	85 ± 8	0.59
HCN2 Control (−100 mV)	840 ± 63	249 ± 28	
30 µM NQTP (−100 mV)	786 ± 72	161 ± 73	0.63
HCN4 Control (−110 mV)	1711 ± 213	177 ± 36	
30 µM NQTP (−110 mV)	2,187 ± 229	350 ± 18	<0.05
HCN1 30 mM [K^+^]_o_ Control (−100 mV)	489 ± 14	89 ± 5	
30 µM NQTP (−100 mV)	613 ± 53	146 ± 18	<0.05
HCN1ΔCNBD Control (−100 mV)	1,240 ± 72	158 ± 7	
30 µM NQTP (−100 mV)	1,520 ± 100	352 ± 40	<0.05

**FIGURE 3:**
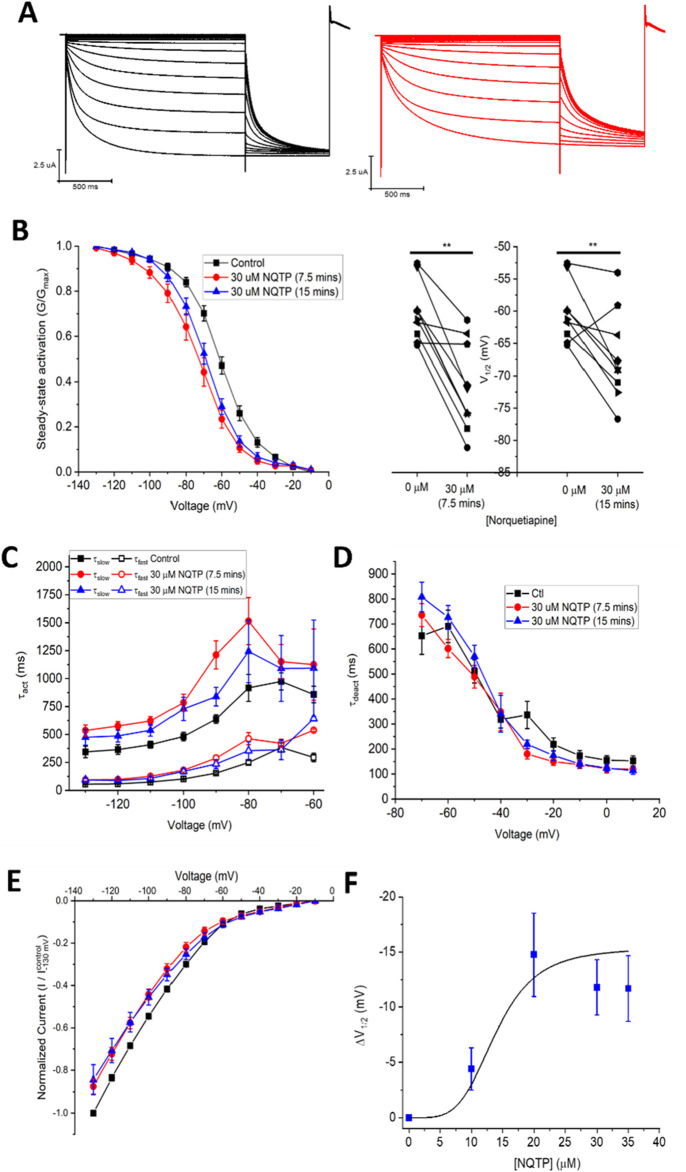
Norquetiapine (NQTP) inhibits HCN1 channels. **(A)** Representative traces from a paired activation experiment (control in black) following the addition of 30 µM NQTP (red traces) to oocytes expressing full-length HCN1. **(B)** NQTP induces a hyperpolarizing shift in the steady-state voltage-dependence of activation (P < 0.05 for V_1/2_ for 7.5 and 15 min compared to control). **(C)** Activation time constants (τ_fast_ and τ_slow_) are greater in the presence of NQTP (P < 0.05). **(D)** Deactivation time constants (τ_deact_) are unchanged in presence of NQTP. (n = 6; P = 0.23). **(E)** Current-voltage (I-V) relationship in presence of NQTP normalized to maximal current (I_Control (−130 mV)_). (n = 9; P < 0.05 for 7.5 and 15 min compared to control). **(F)** Concentration dependence of ΔV_1/2_ fit with a Hill Equation 3 indicates NQTP inhibits HCN1 channels with an IC_50_ of 13.9 ± 0.8 μM, a maximum ΔV_1/2_ of −15.4 ± 1.2 mV and a Hill co-efficient of 4.2 ± 0.1.

**FIGURE 4:**
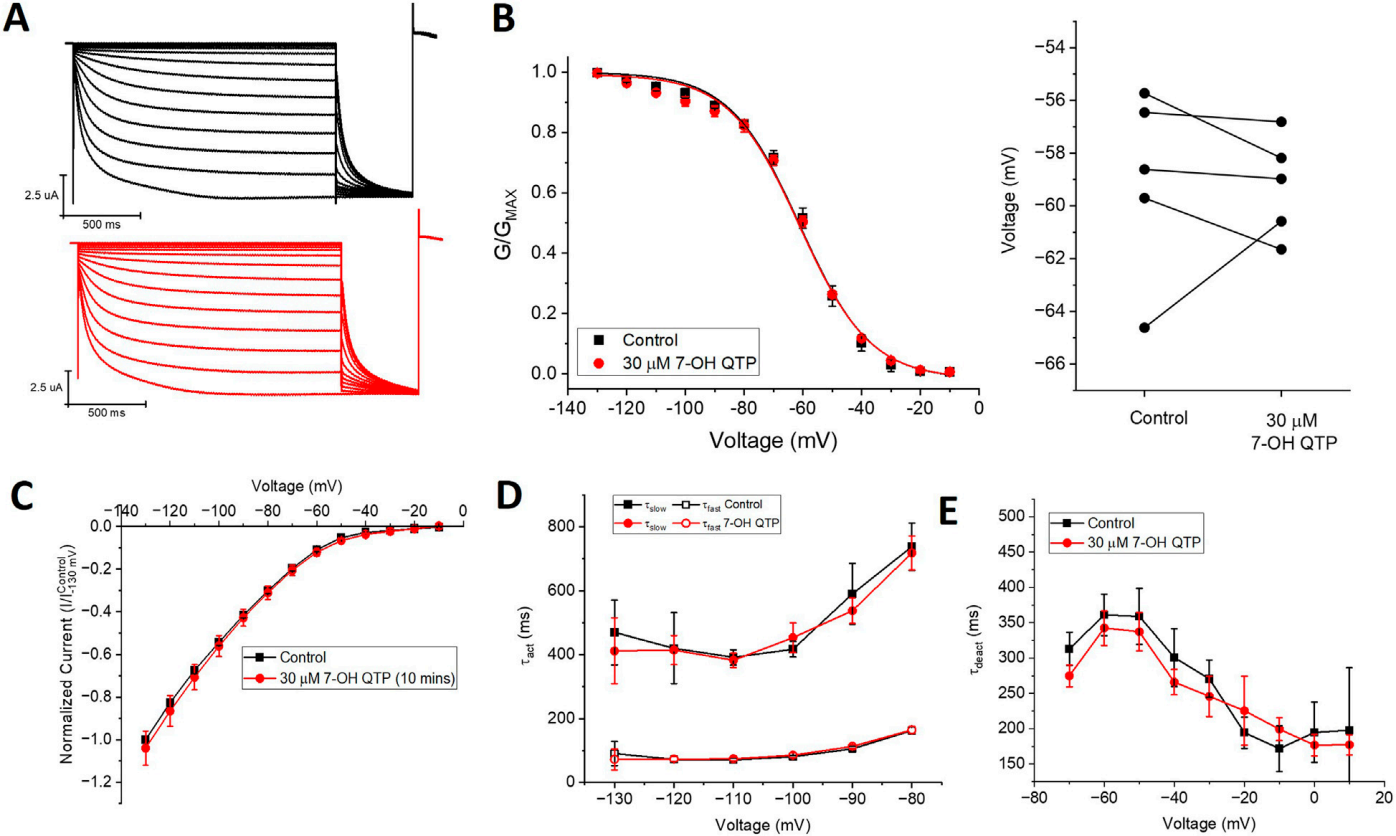
7-hydroxyquetiapine (7-OH QTP) does not regulate HCN1 channels. **(A)** Representative traces from a paired activation experiment (control in black) following the addition of 30 µM 7-OH QTP (red traces) to oocytes expressing full-length HCN1. 30 μM 7-OH QTP does not affect **(B)** the voltage-dependence of activation (V_1/2_ of each cell is shown on the right) (n = 5, P = 0.64) **(C)**, the current-voltage (I-V) relationship (n = 5, p = 0.47), **(D)** activation kinetics (n = 5, p = 0.88), or **(E)** deactivation kinetics (n = 6, P = 0.35) of HCN1 channels.

Since NQTP also exhibits antidepressant activity (Jensen et al., 2008; Lopez-Munoz and Alamo, 2013) and a pharmacological activity that differs from QTP (Bakken et al., 2012; DeVane and Nemeroff, 2001), we also examined the effects of 30 µM NQTP on HCN1 channels (Figure 3). NQTP reduces HCN1 currents (Figure 3E), with a −11.8 ± 0.5 mV hyperpolarizing shift in the voltage-dependence of activation (Figure 3B) and slowing the kinetics of activation (Figure 3C). Deactivation kinetics are unchanged with NQTP treatment (Figure 3D). Examination of the concentration dependence of NQTP on the shift in voltage-dependence of activation (ΔV_1/2_) using Equation 3 indicates an IC_50_ of 13.9 ± 0.8 μM with a maximum ΔV_1/2_ of −15.4 ± 1.2 mV and a Hill co-efficient of 4.2 ± 0.1 (Figure 3F). Contrary to the effects of NQTP on HCN1 function, 7-OH QTP had no observable effects on HCN1 current, voltage-dependence, or gating kinetics (Figure 4) for incubation periods between 10 and 30 min.

### Comparison of inhibition of HCN isoforms by 30 µM norquetiapine

**FIGURE 5:**
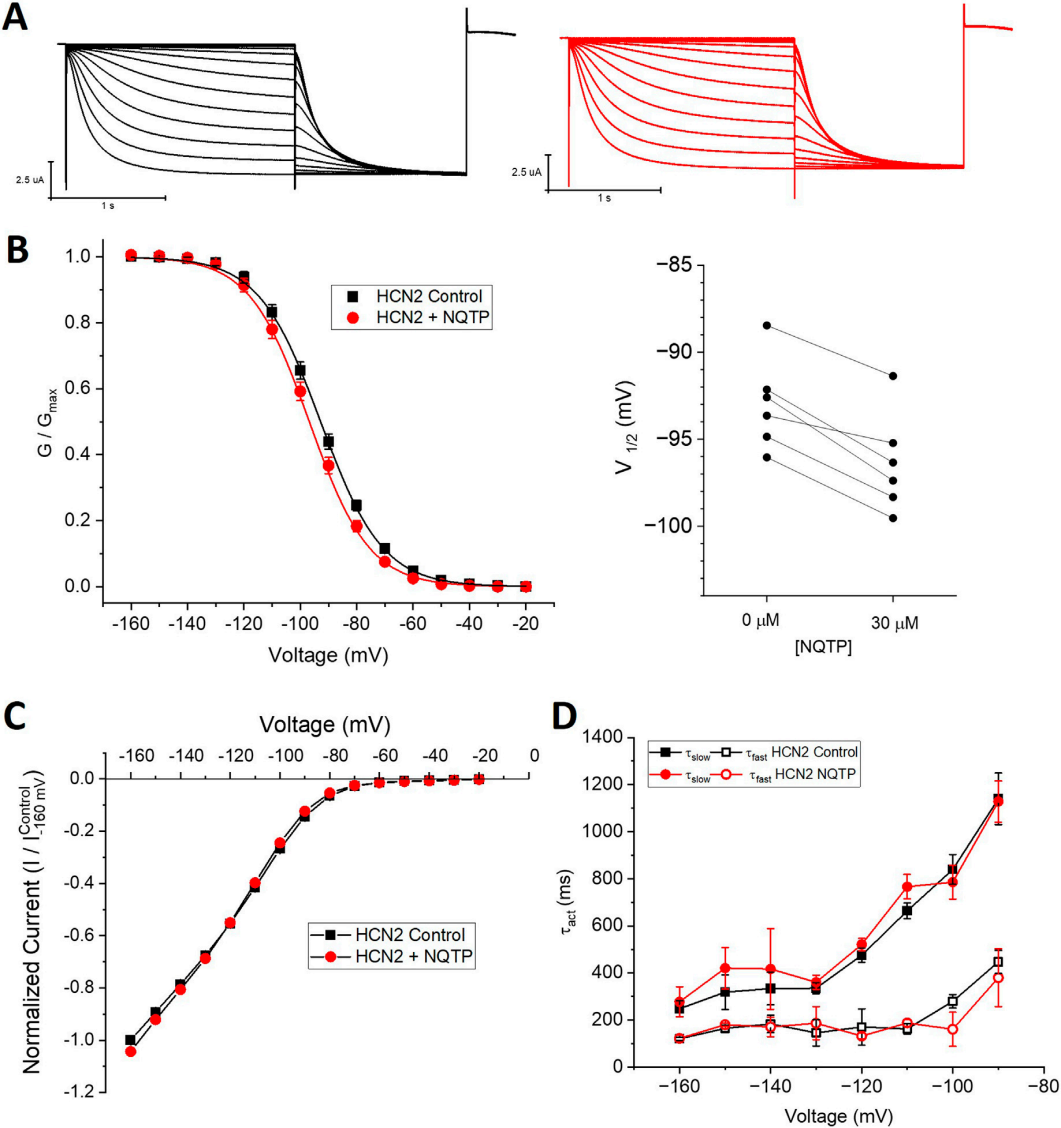
Norquetiapine (NQTP) does not inhibit HCN2 channels. **(A)** Representative traces from a paired activation experiment (control in black) following the addition of 30 µM NQTP (red traces) to oocytes expressing full-length HCN1. Unlike what we observe for HCN1 channels, 30 µM NQTP does not alter **(B)** the voltage-dependence of activation (V_1/2_ of each cell is shown on the right) (n = 6, P = 0.09), **(C)** the current-voltage (I-V) relationship (n = 6, p = 0.79) nor **(D)** the activation kinetics (n = 6, p = 0.65).

**FIGURE 6:**
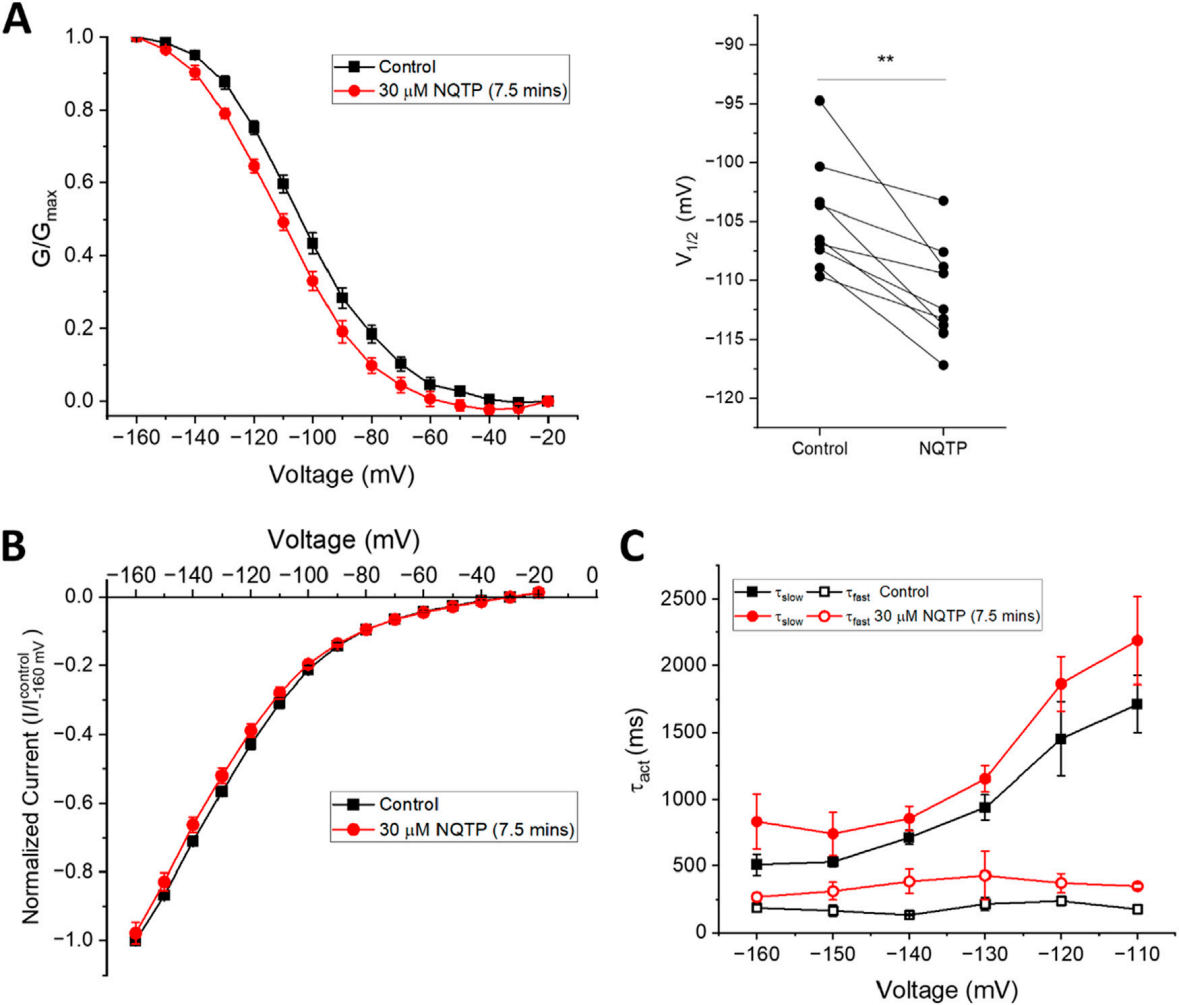
Norquetiapine (NQTP) inhibits HCN4 channels. **(A)** NQTP induces a hyperpolarizing shift in the steady-state voltage-dependence of activation (p < 0.05 for V_1/2_) (V_1/2_ of each cell is shown on the right) (n = 9, p = 0.001). **(B)** Current-voltage (I-V) relationship in presence of NQTP normalized to maximal current (I_Control (-160 mV)_). (n = 9; p = 0.63 for 7.5 min compared to control). **(C)** Activation time constants (τ_fast_ and τ_slow_) are greater in the presence of NQTP (P < 0.05).

To determine if NQTP inhibition is specific to HCN1 channels, we assessed if 30 µM NQTP can also inhibit HCN2 or HCN4 channels. Pair-wised assessment of NQTP on HCN2 leads to a −3.2 ± 1.7 mV change in the voltage-dependence of activation, however, this did not reach statistical significance (P = 0.09). Thus, no significant changes in HCN2 function (I-V relationship, voltage-dependence of activation, or gating kinetics) are observed following incubation with NQTP at concentrations between 10 μM and 30 µM (Figure 5). To ensure the lack of an effect was due to equilibration within the membrane, we extended the period of incubation from 7.5 min to 15 min, however, still no effect is observable. On the other hand, NQTP inhibited HCN4 channels by inducing a −6.5 ± 2.1 mV hyperpolarizing shift in voltage-dependent activation and slowing the rate of activation (Figure 6). Thus, while HCN4 can be inhibited by NQTP, HCN1 channels are inhibited with higher efficacy than HCN2 and HCN4 at the concentrations tested. Notably, in our hands *Xenopus* oocytes do not tolerate the application of >35 µM NQTP and thus a concentration dependence for HCN4 could not be reliably collected.

### Mechanistic characterization of norquetiapine modulation of HCN1 channels

**FIGURE 7:**
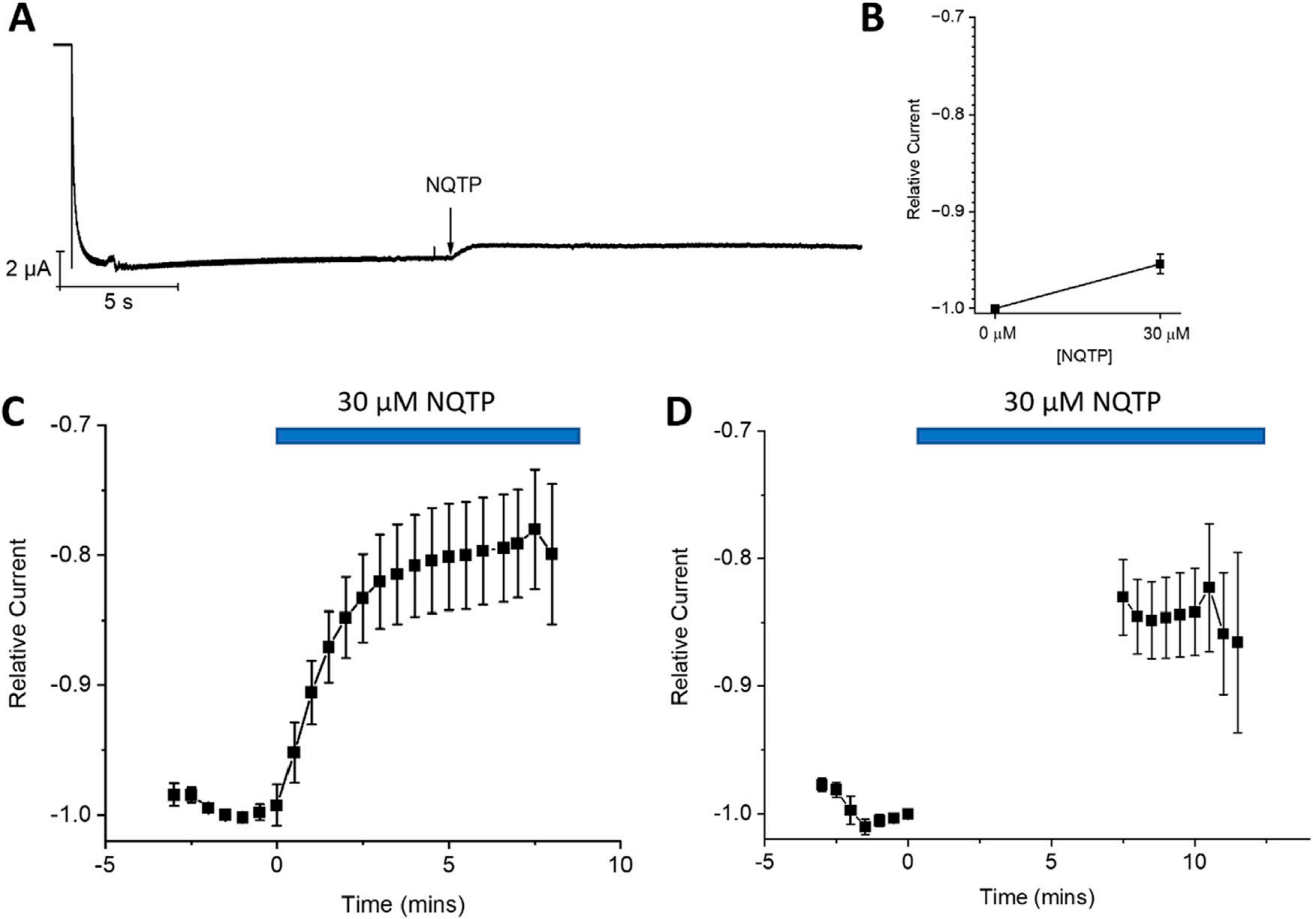
State-dependence of norquetiapine (NQTP) inhibition of HCN1 channels. **(A)** Open-state block was assessed by a prolonged activation step to −130 mV and applying 30 µM NQTP at steady-state. **(B)** Relative current (I_NQTP_/I_control_) to be 0.95 ± 0.01 following NQTP treatment (n = 5; P < 0.05). **(C)** Closed-state block was assessed using a repetitive −130 mV/+30 mV pulse protocol every 30 s 30 μM NQTP was applied after the stabilization of HCN1 currents, and resulted in a decrease of current by 20.4% ± 1.1% (n = 7). **(D)** When the repetitive protocol is interrupted and cells are held at V_H_ = −10 mV for 7.5 min during the application of NQTP the amount of inhibition that is induced is 15.8% ± 0.7% (n = 9) indicating NQTP is a closed state blocker of HCN1.

To assess if NQTP inhibits HCN function from the open state we applied a prolonged activation step to −130 mV, to fully activate the channels, and applied 30 µM NQTP at steady-state (Figure 7A). We observe a current ratio (I_NQTP_/I_control_) of 0.95 ± 0.01 following this protocol. Thus, NQTP has minimal effects on HCN channels in the open state.

We also examined if NQTP inhibition involves interactions in the closed state. We performed a repetitive pulse protocol in which channels were opened at −130 mV for 2 s then closed at +30 mV every 30 s. 30 μM NQTP was applied after the stabilization of HCN1 currents and resulted in a decrease of current by 20.4% ± 1.1% (Figure 7C). This is similar to the amount of inhibition that is induced when the repetitive protocol is interrupted and cells are held at V_H_ = −10 mV for 7.5 min during the application of NQTP (15.8% ± 0.7%) (Figure 7B). Under this condition, cells are predominately closed. Therefore, HCN1 channels can be inhibited by NQTP from the closed state.

**FIGURE 8:**
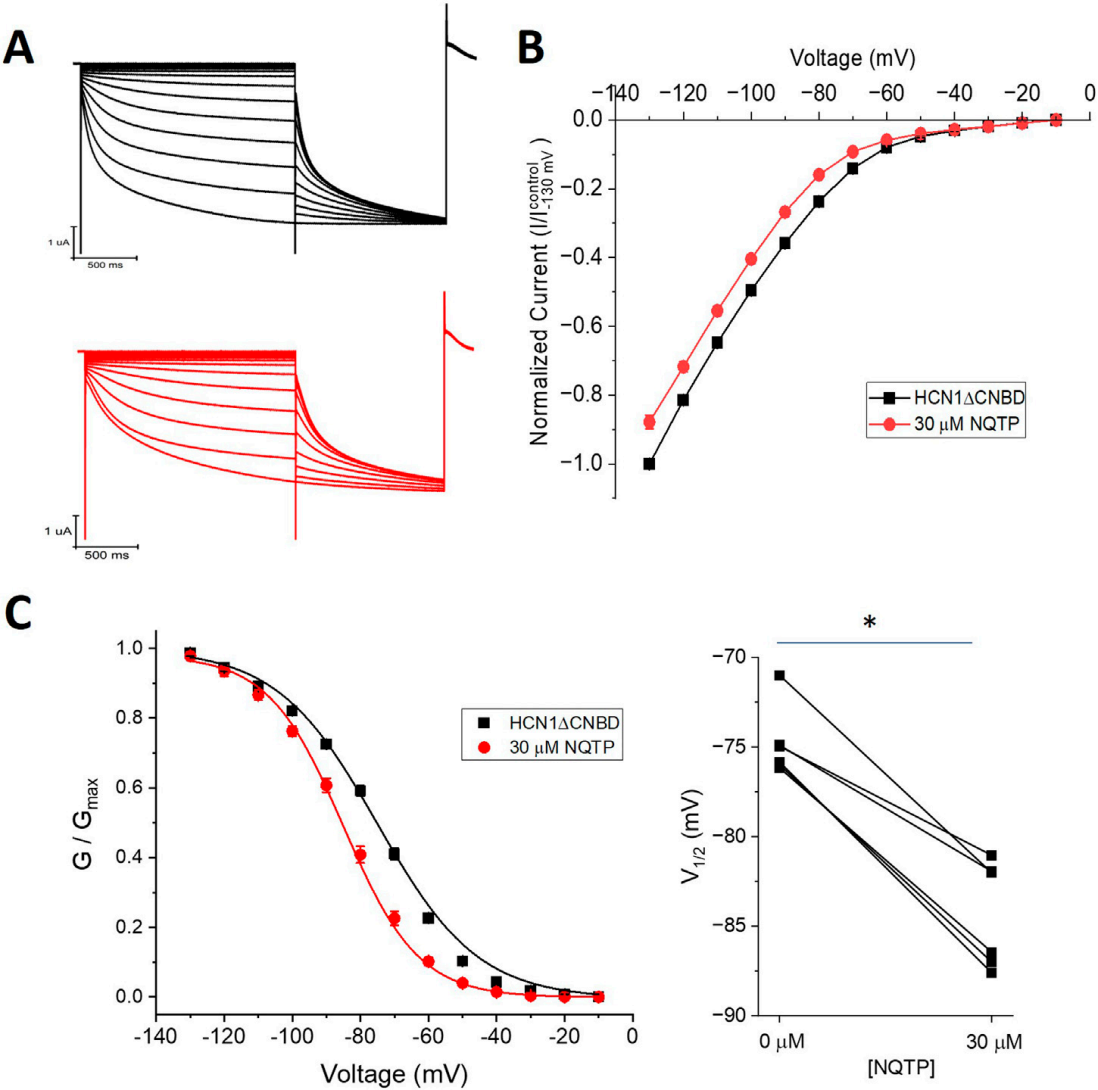
Norquetiapine (NQTP) inhibition of HCN1 channels does not depend on the CNBD. **(A)** Representative traces from a paired activation experiment (control in black) following the addition of 30 µM NQTP (red traces) to oocytes expressing HCN1ΔCNBD. **(B)** Current-voltage (I-V) relationship in presence of NQTP normalized to maximal current (I_control (-130 mV)_). (n = 6; P < 0.05). **(C)** NQTP induces a hyperpolarizing shift in the steady-state voltage-dependence of activation (P < 0.05).

To determine if NQTP acts on HCN1 channels through interactions with the CNBD, we examined the effects on HCN1 channels lacking this domain (HCN1ΔCNBD) (Figure 8). Similarly to full-length HCN1 channels, 30 µM NQTP induces an approximate 10% reduction in the I-V relationship of HCN1ΔCNBD compared to control (Figure 8B). Additionally, 30 µM NQTP shifts the V_1/2_ of activation to more hyperpolarized potentials by −11.8 ± 1.2 mV (Figure 8C). Thus, inhibition of HCN1 channels by NQTP is not dependent on the CNBD.

**FIGURE 9:**
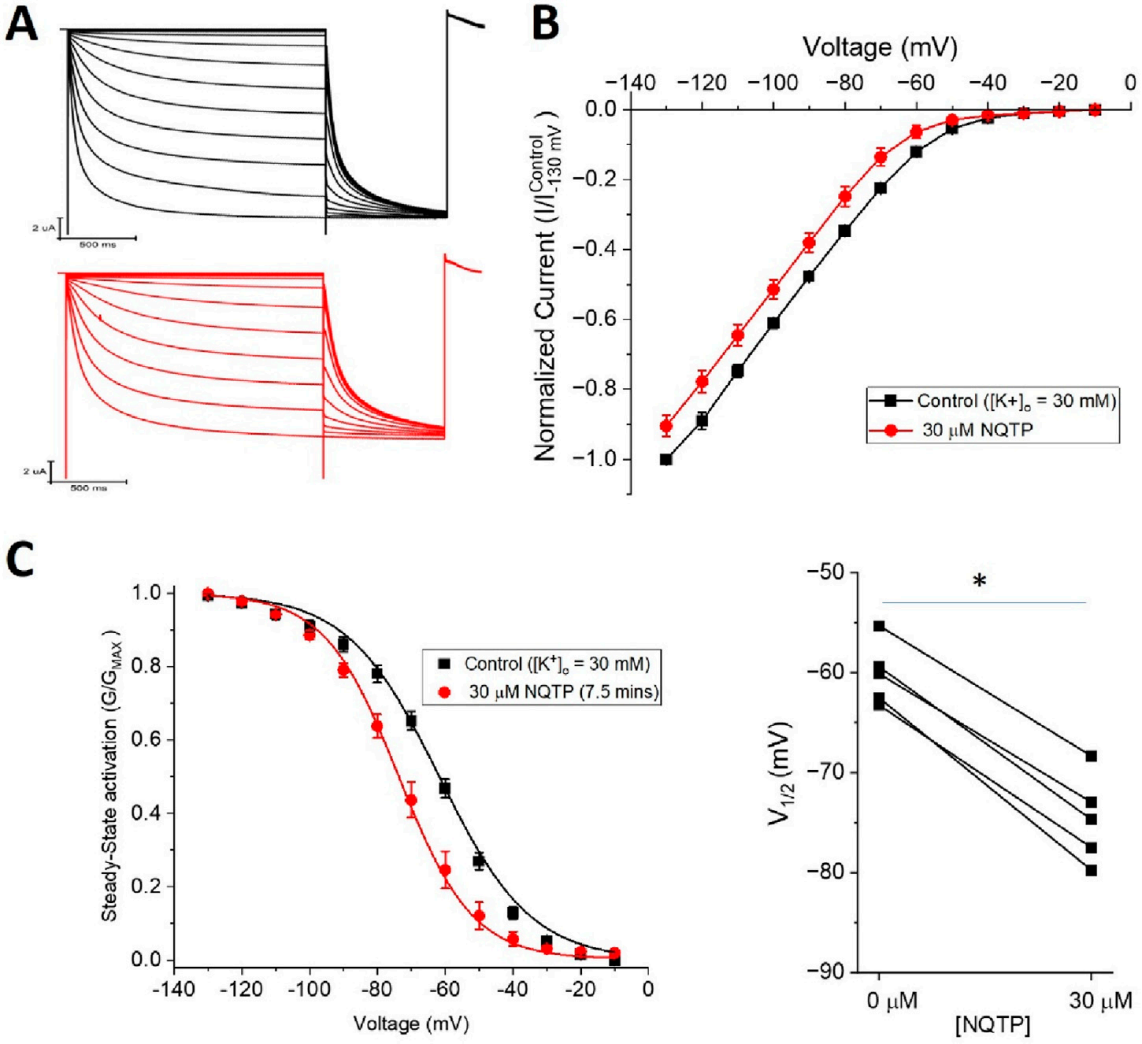
Norquetiapine (NQTP) inhibition of HCN1 channels is not affected by increasing [K^+^]_o_. **(A)** Representative traces from a paired activation experiment following the addition of 30 µM NQTP (red traces) to oocytes expressing full-length HCN1 bathed in high external potassium (control in black). **(B)** Current-voltage (I-V) relationship in presence of NQTP normalized to maximal current (I_control (-130 mV)_). (n = 6; P < 0.05). **(C)** NQTP induces a hyperpolarizing shift in the steady-state voltage-dependence of activation (P < 0.05 for V_1/2_).

If NQTP inhibits HCN channels by binding in the pore region, similarly to ivabradine, ZD7288, clonidine, lidocaine, and other inhibitors (Cheng et al., 2007; Tanguay et al., 2019), we would anticipate that NQTP inhibition would depend on the extracellular K^+^ concentration ([K^+^]_o_). Specifically, we would anticipate that increasing [K^+^]_o_ would reduce the effect of NQTP on HCN1. Instead, we observe that increasing [K^+^]_o_ from 5 mM to 30 mM (by replacing the equivalent amount of extracellular Na^+^) had no effect on NQTP inhibition of HCN1 currents (Figure 9). 30 μM NQTP continues to reduce the I-V relationship (Figure 9B), and induces a hyperpolarizing shift in the V_1/2_ of activation by −9.5 ± 0.4 mV (Figure 8C) and slowed activation of HCN1 in 30 mM [K^+^]_o_. Thus, these data suggest that NQTP inhibition occurs via a different mechanism than many other known inhibitors of HCN channels which block the pore-domain and are sensitive to [K^+^]_o_.

## Discussion

Antipsychotics are a class of psychotropic medication primarily used to manage psychosis (including delusions, hallucinations, paranoia or disordered thought), principally in schizophrenia and bipolar disorder. All antipsychotics block dopamine D2 receptors, however, “atypical” antipsychotics (AAPs) bind less avidly to D2 receptors, leading to fewer extrapyramidal side effects at appropriate doses. AAPs also antagonize serotonin receptors, mainly 5HT2A. Consequently, AAPs are being used more often in recent years to treat anxiety, MDD, PTSD and other disorders. Quetiapine (QTP), a second-generation AAP commonly prescribed for the treatment of schizophrenia (Small et al., 1997; Dev and Raniwalla, 2000) and acute bipolar mania (Janicak and Rado, 2012), is now also used to treat insomnia (Lin et al., 2023), MDD (Ravindran et al., 2022), anxiety (Ravindran et al., 2022; Crapanzano et al., 2021; Bandelow et al., 2010), and PTSD (Crapanzano et al., 2023). Like other AAPs, QTP is structurally similar to clozapine and acts as an antagonist to serotonin, dopamine, histamine, and adrenergic receptors (Saller and Salama, 1993; Burns, 2001). QTP is primarily metabolized by hepatic cytochrome P450 3A4 (Dev and Raniwalla, 2000), with norquetiapine (NQTP) as its major active metabolite (Figure 1). NQTP exhibits pharmacological activity that differs from QTP (Bakken et al., 2012; DeVane and Nemeroff, 2001) and also exhibits antidepressant activity (Jensen et al., 2008; Lopez-Munoz and Alamo, 2013). In fact, NQTP shares structural similarities with several antidepressants including amoxapine and desipramine, and its physicochemical properties confer greater potential for its use as an antidepressant agent (Lopez-Munoz and Alamo, 2013; Kim et al., 2016). Indeed, the effect of QTP in major depressive disorder is probably mediated, at least in part, by NQTP, which selectively inhibits norepinephrine transporter reuptake (Bandelow et al., 2010; Lopez-Munoz and Alamo, 2013). Given the role of HCN channels in MDD, anxiety, and schizophrenia, we hypothesize that QTP and its major metabolites NQTP and 7-OH may work effectively in part via inhibition of HCN function.

Here we demonstrate that norquetiapine, but not quetiapine, inhibits HCN1 channels by shifting the voltage-dependence to hyperpolarized potentials and slowing channel opening. NQTP inhibited HCN1 channels with an IC_50_ of 13.9 ± 0.8 μM. This is similar to the ranges in which QTP and NQTP were found to inhibit other ion channels. Specifically, QTP and NQTP were found to block hERG current with a half-maximal inhibitory concentration of 8.3 and 10.8 μM, respectively (Lee et al., 2018). Nav1.5 currents were also shown to be inhibited by QTP and NQTP with IC_50_ of 30 and 6 μM respectively (Kim et al., 2020). Notably, HCN1 currents are still inhibited, with an approximate −3 to −7 mV shift in activation at these concentrations (Figure 3F). It is speculated that the inhibition of cardiac sodium channels by these drugs can reduce the risk of cardiotoxicity induced by the inhibition of hERG current. By comparison, inhibition of HCN channels by other molecules including ivabradine, ZD7288, clonidine, lidocaine, ketamine, and carvedilol also act within a similar concentration range (Cheng et al., 2007; BoSmith et al., 1993; Bucchi et al., 2006; Cao et al., 2018; Putrenko et al., 2017; Xing et al., 2017). Therefore, inhibition of HCN channels by NQTP occurs within the physiological range, and likely contributes to its therapeutic role.

It is not uncommon that for a number of drugs acting on a given target, the EC_50_/IC_50_ determined in oocyte experiments differ from EC_50_/IC_50_ determined in mammalian cells, however, the underlying effects remain. For example, the IC_50_ of hERG channel block by the antiarrhythmic drug BRL-32872 is ∼240 nM in *Xenopus* oocytes, but ∼20 nM for mammalian HEK293 cells (Thomas et al., 2001). Acehytisine blocks HCN4 channels expressed in *Xenopus* oocytes with an IC_50_ of ∼65 μM, but ∼10 µM in native I_f_-channels of the rabbit SAN (Fan et al., 2012). Furthermore, the IC_50_ of ketamine inhibition of HCN1 expressed in HEK293 cells is ∼8 μm for V_1/2_ and ∼16 μm for current amplitude (Chen et al., 2009), but 67 μM in *Xenopus* oocytes (Xing et al., 2017). Thus, it is possible, or even likely, that NQTP effects HCN1 and HCN4 channels at even lower concentrations in mammalian cells.

Inhibition of HCN1 channels has been suggested as a key therapeutic target for depression. HCN1^−/−^ mice show impaired motor learning but enhanced spatial learning and memory (Nolan et al., 2004; Nolan et al., 2003) and enhanced resistance to depression (Lewis et al., 2011; Huang et al., 2009). Targeted knockdown of HCN1 by shRNA in the CA1 hippocampal region enhances mobility in the Porsolt swim test, a behavioural model for anti-depressant effects (Kim et al., 2012). Furthermore, HCN1 expression increases in the CA1 region of the dorsal hippocampus of chronic unpredictable stress rats, while stress responses are reduced upon HCN1 shRNA knockdown (Kim et al., 2018). Additionally, *Trip8b* knockout mice are also more resistant to depression (Lewis et al., 2011). Recently, the benzisoxazole derivative Org 34,167, which has been patented for the treatment of depression and progressed to Phase I trials, was shown to be a broad-spectrum brain penetrant inhibitor of HCN channels, and resulted in reduced marble burying and increased the time spent mobile in the Porsolt swim and tail suspension tests in both male and female mice, suggesting reduced depressive-like behaviour (Pinares-Garcia et al., 2023). Thus, the inhibition of HCN1 channels by norquetiapine could provide a therapeutic benefit and contribute to the molecular mechanism of its anti-depressant action. Furthermore, the ability for HCN1 channels to be inhibited by NQTP but not QTP or 7-OH QTP may also provide a platform from which to develop even more specific negative gating modulators of HCN channels.

NQTP selectivity for HCN1 over HCN2 and HCN4 may also be an important factor in its therapeutic role. Intriguingly, the role of HCN2 and HCN4 in major depressive disorder is less straightforward than HCN1. HCN2^−/−^ mice spend more time mobile in the tail suspension test (Lewis et al., 2011), suggesting a reduction in HCN2 has antidepressant effects. However, the expression of HCN2 is reduced in cholinergic interneurons in the nucleus accumbens of mice subjected to chronic stress, while HCN2 overexpression rescues the depressive phenotypes (Cheng et al., 2019). Similarly, HCN2 overexpression in dopamine neurons of the ventral tegmental area is effective in reversing the depressive phenotypes caused by chronic mild unpredictable stress in mice (Zhong et al., 2018). These data suggest that unlike what was observed for HCN1, stimulation of HCN2 is more favourable for the treatment of depression than HCN2 inhibition. Similarly, while HCN4 knockdown in the hippocampus suggested an increase in anxiety-like activity (Gunther et al., 2019), brain-specific HCN4 knockdown had a subtle anxiolytic effect (Kharouf et al., 2020). Thus, further supporting a role for NQTP inhibition of HCN1, and possibly HCN4, as part of the therapeutic mechanism of action of NQTP. However, since HCN4 channels also play a critical role in cardiac rhythmicity, examinations into the bradycardic effects of future NQTP-based treatments should also be considered.

HCN channels are blocked by a number of inhibitors, with ivabradine arguably the best characterized. Ivabradine does not demonstrate isoform specificity between HCN1-4 channels (Bucchi et al., 2006; Stieber et al., 2006). Ivabradine blocks the open state of HCN4 when the channels are opened by hyperpolarization, with enhanced binding upon frequent changes in the direction of ion flow (Bucchi et al., 2006; Bois et al., 1996; Bucchi et al., 2002) However, HCN1 channels can also be inhibited from the closed state (Bucchi et al., 2006). ZD7288 is also an open-state blocker of HCN channels (Cheng et al., 2007; Benetos et al., 1999; Shin et al., 2001; Wu et al., 2012) that induces at −15 mV shift in voltage-dependent I_h_ activation and reduces maximal activity by more than 50% (BoSmith et al., 1993). Lidocaine, bupivacaine and mepivacaine blockade of HCN channels also occurs from the inside of the cell (Putrenko et al., 2017). These inhibitors may bind in the open pore interacting with residues C358, A383, Y386, A387, V390 (HCN1 numbering) with residues C358, Y386, and A387 lining a hydrophobic groove within the pore cavity that may conformationally restrict the smaller ligands (Tanguay et al., 2019). Notably, these inhibitors are sensitive to extracellular potassium concentrations. Thus, it does not appear that NQTP inhibition of HCN1 follows the same pore-binding mechanism as these inhibitors.

On the other hand, other HCN inhibitors identified appear to act primarily as negative gating modulators of HCNs, rather than blockers of the ion conduction pathway. Niflumic acid may interact with the outer voltage-sensor domain (Cheng and Sanguinetti, 2009) While the binding site of carvedilol is not yet resolved, it is a closed-state inhibitor of HCN channels that induces a hyperpolarizing shift in the voltage-dependence of activation, but interacts with the channels at a site distinct from the pore-binding site of ivabradine or ZD7288 (Cao et al., 2018). There is also no evidence that ketamine, which inhibits HCN1 channels with a −20 mV shift in voltage-dependence and a reduction in activation kinetics at 25 μM, but not HCN2 and HCN4 at that concentration, could act as a pore inhibitor (Xing et al., 2017). Our data indicates that NQTP acts to inhibit HCN1 more like a negative gating modulator, rather than a pore blocker, since it modulates the voltage-dependence and gating kinetics of activation, is a closed-state blocker, and insensitive to external potassium concentration.

Our findings that NQTP is a selective inhibitor of HCN1 channels contributes to the understanding of the mode of action of quetiapine for the treatment of neuropsychiatric disorders such as anxiety, major mood disorder, and others. Our results may assist in the development of improved therapeutics based on this molecular scaffold.

## Data Availability

The raw data supporting the conclusions of this article will be made available by the authors, without undue reservation.

## Abbreviations

MMD, major mood disorder; HCN, hyperpolarization-activated cyclic-nucleotide gated; CNBD, cyclic-nucleotide binding domain; WT, wild-type; FL, full-length; QTP quetiapine; NQTP, norquetiapine.
